# Supplementary material for: A distributive peptide cyclase processes multiple microviridin core peptides within a single polypeptide substrate
Source: Nat Commun. 2018 May 3;9:1780. doi: 10.1038/s41467-018-04154-3 (PMC5934393; doi:10.1038/s41467-018-04154-3)
Supplement: Supplementary file 2 — Supplementary Information [file 41467_2018_4154_MOESM2_ESM.pdf]

Supplementary Information

A distributive peptide cyclase processes multiple microviridin core peptides within a single polypeptide substrate

Zhang et al.

Supplementary Methods

1. Reagents and materials. All chemicals were purchased from Sigma Aldrich or Fisher Scientific unless noted otherwise. Endoproteinases (GluC) and Q5 High-Fidelity DNA polymerase were purchased from New England BioLabs, Inc. T4 DNA ligase, dNTP solutions, all restriction endonucleases, Gel extraction and PCR purification kits were purchased from Thermo Fisher Scientific. Primers were ordered from Sigma-Aldrich. Polymerase chain reaction (PCR) amplifications were carried out using an automated thermocycler (Eppendorf 5344 Mastercycler ep 384). *Escherichia coli* DH5α, BL21-GOLD (DE3), BL21-C43 (DE3) (Lucigen) were used for routine molecular biology studies and protein expression, respectively, and were grown in Luria-Bertani broth or Terrific broth. DNA sequencing was performed at Eurofins. Plasmid miniprep kits were purchased from Zymo Research. A Shimadzu Prominence UHPLC system (Kyoto, Japan) fitted with an Agilent Eclipse Plus C18 column (3.5 μm, 2.1 x 100 mm, 90Å), coupled with a PDA detector was used for HPLC analysis. A 3200 QTRAP (Applied Biosystems) equipped with a Shimadzu UPLC system was used for low-resolution LC-MS analysis in the studies. High resolution LC-MS (HR-LC-MS) and LC-MS/MS (HR-LC-MS/MS) analyses were conducted using a Thermo Fisher Q Exactive Focus mass spectrometer equipped with UltiMate™ 3000 RSLCnano System and electrospray probe on Universal Ion Max API source. Thermo Xcalibur Roadmap™ was used to process HR-LC-MS data. Protein binding affinities were measured by a ForteBio Octet® RED384 System equipped with ForteBio Dip and Read™ Anti-Penta-HIS1K (HIS1K) Biosensors. Chemical structures were drawn using ChemBioDraw Ultra. Homology modeling and structure rendering were performed using Discovery Studio 2.5.

2. Bioinformatic mining of microviridin gene clusters. To identify microviridin gene clusters, BlastP searches were performed using the AMdnA protein sequence as the query. The individual adjacent open reading frames (ORFs) were manually inspected with the Conserved Domain Database analysis to identify putative ATP grasp ligases and other enzymes involved in microviridin biosynthesis (e.g., MdnD analogs for *N*-acetylation and MdnE analogs for cross-membrane transportation).

3. Multiple sequence alignment for AMdnA and AMdnC analogs. Since the biosynthesis of microviridins has been previously characterized in microviridin B, J, K and L systems, the nucleotide and amino acid sequences of AMdnC analogs, their precursor peptides were retrieved from the National Center for Biotechnology Information (NCBI) sequence database. Multiple sequence alignment was conducted using a web server T-Coffee[1] and visualized using Jalview Version 2[2].

4. Heterologous expression and purification of recombinant proteins. Expression constructs were transformed into *E. coli* C43 (DE3) competent cells for protein expression. Transformed cells were cultured in Terrific Broth medium supplemented with kanamycin (50 μg/ml). For the expression of AMdnC and mutants, the plasmid pGro7 was co-transformed. In these cases, cells were cultured in Terrific Broth medium supplemented with kanamycin (50 μg/ml), chloramphenicol (25 μg/ml) and L-arabinose (1 mg/ml) according to the manufacture's protocol (Takara). Cultures were grown at 37 °C, 250 rpm

until OD600 reached 0.6. Protein expression was then induced by isopropyl-β-D-thiogalactopyranoside (IPTG) at a final concentration of 0.1 mM at 16 °C, 250 rpm for 16 h. After centrifugation (5,000 g, 15 min, and 4 °C), cell pellets were collected and stored in -80 °C or directly used for protein purification. Protein purification followed our previous protocol[34]. Briefly, cell pellets were first suspended in the proper volumes of lysis buffer (cell biomass: buffer = 1:4, v:v) [100 mM Tris-Cl, pH 8.0, 100-500 mM NaCl, 5-20 mM imidazole, 3 mM β-mercaptoethanol (BME) and 10 % glycerol]. A protease inhibitor cocktail containing Leupeptin (10 μM), Pepstatin (1 μM), PMSF (1 mM) and EDTA (5 mM) was supplemented to the lysis buffer for the purification of AMdnA, its mutants and variants. Soluble proteins were released by sonication and collected by centrifugation at 35,000 x g at 4 °C for 30 min. Recombinant proteins were purified using Ni-NTA agarose resin (Thermo), and eluted in the lysis buffer with 50 to 300 mM imidazole. After SDS-PAGE analysis, elution fractions containing the targeted proteins were combined. The proteins were then exchanged into a storage buffer (25 mM Tris-HCl, pH 8.0, 100 mM NaCl, 3 mM BME, and 10 % glycerol) using a PD-10 column according to the manufacture's protocol (GE), aliquoted and stored at -80 °C until the use. For biochemical characterization, the targeted proteins were concentrated using Milli-pore™ Amicon™ Ultra-0.5 and Ultra-2.0 Centrifugal Filter Unit (Millipore). Protein concentration for AMdnA variants and GroEL was determined using the Bio-Rad Bradford Protein Assay. For AMdnC and its mutants, the total protein concentrations were measured first following Bradford Protein Assay protocol, then the concentrations of target proteins were obtained by densitometry based on SDS-PAGE. The band intensities were analyzed by Bio-Rad Image Lab™ Software and the relative quantities calculated by the software were used to give corrected protein concentrations. Purified AMdnA, mutants, and variants were analyzed by LC-HR-MS, and several modified AMdnA species were detected at the low levels (**Figs. 2a**, **4**, **6** and **7**, **Supplementary Fig. 19**). Compared to intact AMdnA, these species were likely the products of putative dehydration, oxidation and/or other unknown modifications during overexpression in *E. coli*. At the finite concentrations, they showed no effects on the overall reaction of AMdnC.

5. Reduction of macrolactonizated AMdnA. The AMdnC reaction was performed as described above. $LiBH_4$ (4.5 mg) in methanol (250 μL) was added to the reaction mixture (100 μL), which was stirred at room temperature. After 30 min, glacial acetic acid (approximately 25 μL) was used to quench the reaction. The reaction mixture was then dried under vacuum. The resulted solid was dissolved in 50 μL methanol/water (1:1) for LC-HR-MS analysis as described above.

6. Proteomic analysis to determine the ring topology of processed AMdnA and its mutants. The reactions were set up as described above and stopped at 0.5 h, 2 h, and 16 h by heating at 95 °C for 5 min. Reaction mixture (50 μL) was then cooled to room temperature, mixed with 50 μL of the digestion buffer of GluC and incubated with GluC [NEB, 1:10 (w/w) protease: protein] at 37°C for 16 h. The proteolytic reaction was quenched with an equal volume of methanol. The resulting mixture was analyzed by LC-HR-MS as described above.

7. Determination of binding affinities between peptide substrates and AMdnC. ForteBio Octet® RED384 System equipped with ForteBio Dip and Read™ Anti-Penta-HIS1K (HIS1K) Biosensors was used to quantitate the binding between AMdnA and its analogs and AMdnC. The biosensors were hydrated in PBS buffer (pH 7.4) for 10 min prior to the experiment. AMdnC was reconstituted in PBS buffer to the concentration of 0.05 mg/mL. The concentrations of analytes were varied from 2.3 to 37 μM. The time scheme setting was as follows: initial baseline for 200 s, loading for 900 s, baseline for 600 s, association for 900 s, and dissociation for 900 s. Correction of baseline drift was performed by subtracting the averaged shift recorded for a sensor loaded with AMdnC but incubated with PBS buffer. Experimental data were fitted using a global fit 1:1 model to determine the K_D values.

8. Homology modeling of AMdnC. A crystal structure of MdnC (PDB code 5IG9) was used as the crystallographic coordinate template. Homology modeling of AMdnC was performed based on the reference protein model using Protein Modeling module embedded in Discovery Studio 2.5. The optimized model was evaluated by the Ramachandran plot analysis.

Supplementary Table 1. Cloning and mutagenesis primers used in this study.

Primer names	Sequence (5' to 3')
AMdnC-FW	GCGGATCCATGAATGTCTTAATTATTACTCACAGCCACG
AMdnC-RV	GAGCGGCCGCTCAAATATGACTTAGTAAAACTTTAGCGATCGC
K165A-FW	CAACAGGATGTAATTACAGCAATGCTTTCTTCCTTTGCC
K165A-RV	GGCAAAGGAAGAAAGCATTGCTGTAATTACATCCTGTTG
D280A-FW	GGTTTAAATTACGGGGCAATTGCGGTAATTTTAACCCCAGATA ATCG
D280A-RV	CGATTATCTGGGGTTAAAATTACCGCAATTGCCCCGTAATTTAA ACC
AMdnA-FW	GCGCATATGATGCCAGAGAATAGACAAGAAG
AMdnA-RV	GAGCGGCCGCCTAACCAACTGGTTGATCATC
D51A-FW	CAAGAAAGTATCCTTCTGCGTGTGAAGATGGTAATGGTG
D51A-RV	CACCATTACCATCTTCACACGCAGAAGGATACTTTCTTG
E53A-FW	GTATCCTTCTGATTGTGCAGATGGTAATGGTGTAAC
E53A-RV	GTTACACCATTACCATCTGCACAATCAGAAGGATAC
D76A-FW	CTCTGAAGTATCCTTCTGCCAATGAAGACAATGGTGG
D76A-RV	CCACCATTGTCTTCATTGGCAGAAGGATACTTCAGAG
E78A-FW	GTATCCTTCTGATAATGCAGACAATGGTGGTGGC
E78A-RV	GCCACCACCATTGTCTGCATTATCAGAAGGATAC
D93A-FW	CTGAAATTTCCATCTGCTGACGATGATCAACCAG
D93A-RV	CTGGTTGATCATCGTCAGCAGATGGAAATTTCAG
D95A-FW	GAAATTTCCATCTGATGACGCTGATCAACCAGTTGGTTAG
D95A-RV	CTAACCAACTGGTTGATCAGCGTCATCAGATGGAAATTTC
T59A-FW	GAAGATGGTAATGGTGTAGCGGGGAAACTTCGTGATGAAG
T59A-RV	CTTCATCACGAAGTTTCCCCGCTACACCATTACCATCTTC
AMdnAc-FW	GCGCATATGCCAGAGAATAGACAAGAAG
AMdnAc-RV	ATACTCGAGCTAACCAACTGGTTGATCATC
M1V1-RV	GACAGCAATGTCTTCATCAC
M1V2-RV	ACCATCTTCACAATCAGAAGG
M1M2c-RV	ATACTCGAGCTAACCACCATTGTCTTCATTATC
M123M3-RV	ATACTCGAGCTATTGATCATCGTCATCAGATGGAAATTTCAGA GTAACAATTTCGCCACCAACTGGTTGATCATC
13L-M2M3V1-RV	CGGTTACACCATTACCATCTCCACCACTAACTGCTTCTG
13L-M2M3V2-RV	CAGAGTGACAGCAATGTCTCCACCACTAACTGCTTCTG
M2M3V1-FW	GATGGTAATGGTGTAACCG
M2M3V1-FW	GACATTGCTGTCACTCTG

Supplementary Table 2. Amino acid sequences of AMdnA and its select variants used in this study

Names	Sequence (*N* to *C*)
AMdnA with *N*-His$_6$ tag	GSSHHHHHHSSGLVPRGSHMMPENRQEDLNAQAVPFFARFLEGQN CEDLTDEESEAVSGGKRGQTRKYPSDCEDGNGVTGKLRDEDIAVTL KYPSDNEDNGGGEIVTLKFPSDDDDQPVG
AMdnA	GSHMMPENRQEDLNAQAVPFFARFLEGQNCEDLTDEESEAVSGGK RGQTRKYPSDCEDGNGVTGKLRDEDIAVTLKYPSDNEDNGGGEIVTL KFPSDDDDQPVG
AMdnA with *C*-His$_6$ tag	PENRQEDLNAQAVPFFARFLEGQNCEDLTDEESEAVSGGKRGQTRK YPSDCEDGNGVTGKLRDEDIAVTLKYPSDNEDNGGGEIVTLKFPSDD DDQPVGLEHHHHHH
AMdnAi	GSHMMPENRQEDLNAQAVPFFARFLEGQNCEDLTDEESEAVSGGK RGQTRKYPSACEDGNGVAGKLRDEDIAVTLKYPSANEDNGGGEIVTL KFPSADDDQPVG
M1V2	GSHMPENRQEDLNAQAVPFFARFLEGQNCEDLTDEESEAVSGGKR GQTRKYPSDCEDG
M2M3V2	GSHMPENRQEDLNAQAVPFFARFLEGQNCEDLTDEESEAVSGGDIA VTLKYPSDNEDNGGGEIVTLKFPSDDDDQPVG

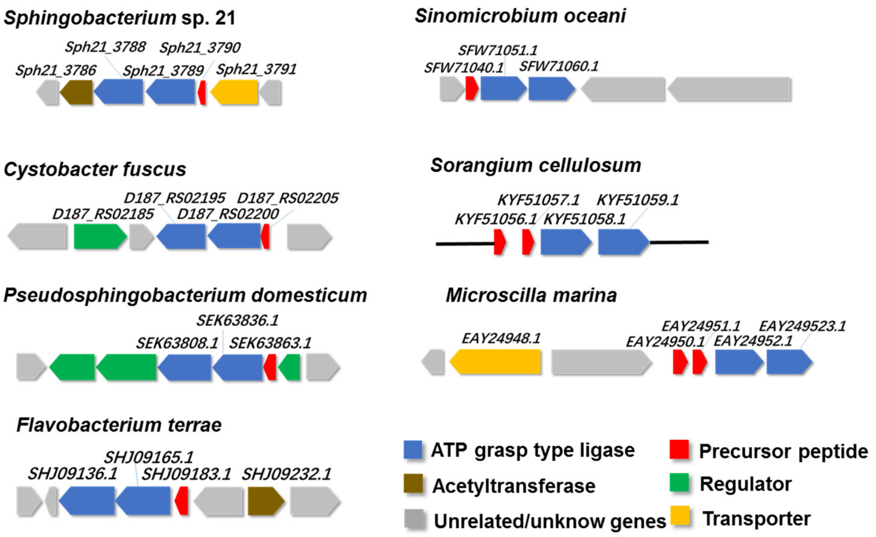

Supplementary Figure 1. Graphical representation of putative microviridin gene clusters from various microbes beyond the phylum of cyanobacteria. The results were obtained from NCBI database by BLAST homology search using AMdnA as the query. Block arrows represent open reading frames (ORFs); the accessible numbers of the ORFs are indicated above the arrows; the sizes of genes are drawn to their scale.

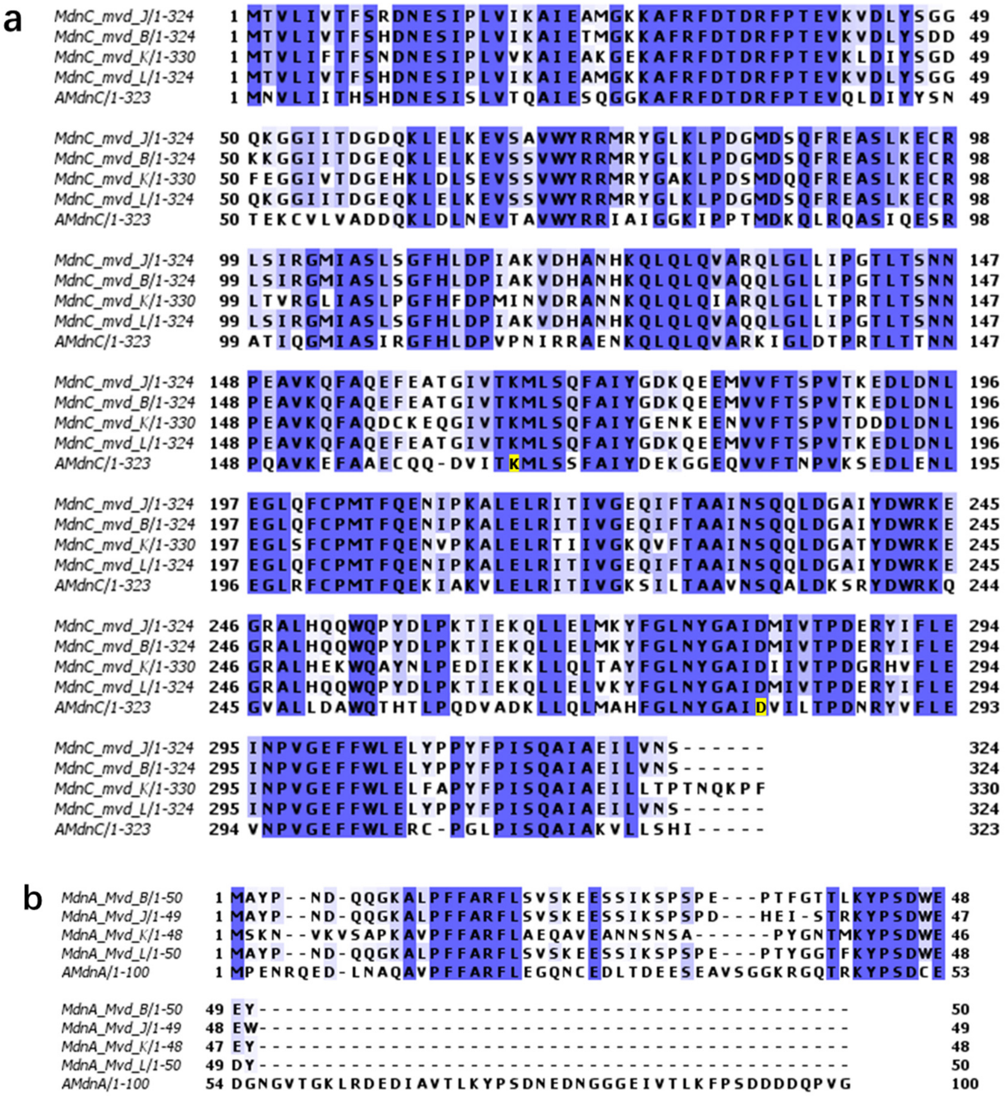

Supplementary Figure 2. AMdnC and AMdnA share sequence identities with their corresponding homologs. a Sequence alignment of AMdnC homologs in various microviridin biosynthetic pathways. AMdnC exhibits 61%, 62%, 63% and 61% identity with its homologs in microviridin B, J, K and L systems, respectively. K165 and D280 of AMdnC were conserved among all homologs, and highlighted in yellow. **b** Sequence alignment of AMdnA homologs in various microviridin biosynthetic pathways. Compared to previously reported precursor peptides of microviridin, AMdnA contains two more core peptides at its *C* terminus.

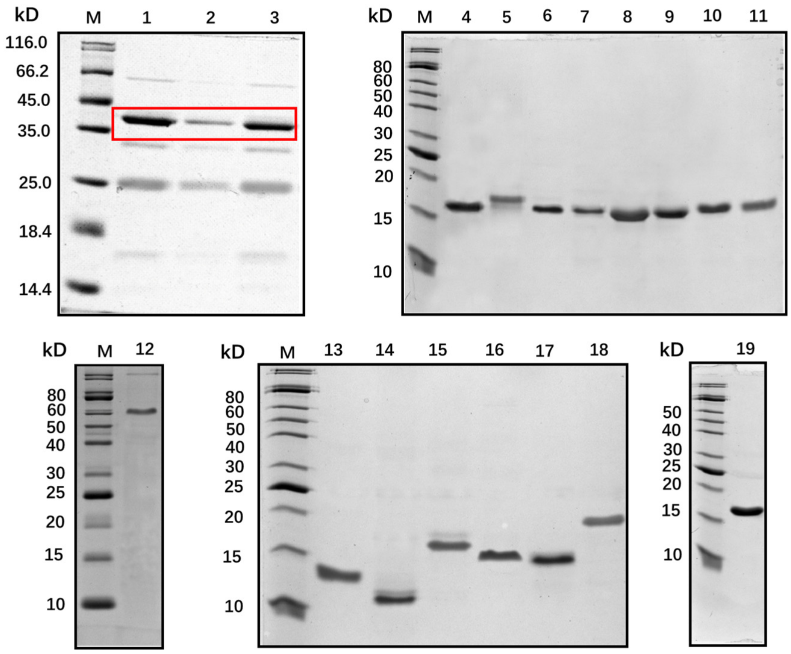

Supplementary Figure 3. SDS-PAGE of purified recombinant proteins used in this study. M: protein marker; Lane 1-3: AMdnC (~ 39 kD), K165A, D280A; lane 4-11: AMdnA (~ 14 kD), AMdnA with C-His_6 tag, D51A, E53A, D76A, E78A, D93A and D95A; lane 12: GroEL (~ 60 kD); lane 13-18: M1V1 (~ 10 kD), M1V2 (~ 8 kD), M2M3V1 (~ 11 kD), M2M3V2 (~ 10 kD), M1M2 (~ 10 kD), M123M3 (~15 kD), lane 19: AMdnAi (~ 14 kD). The molecular weights of some recombinant AMdnA variants were different with their calculated values but the identities of all of these proteins were confirmed in the HR-MS analysis.

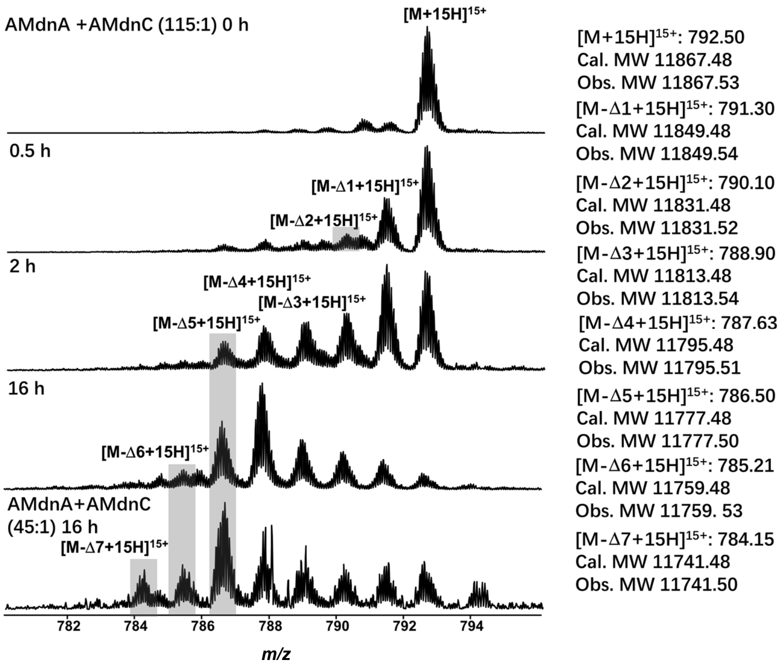

Supplementary Figure 4. AMdnC processed the AMdnA with a *C*-His$_6$ tag. The most abundant species in the reaction contained 5 dehydrations and an increased substrate/enzyme molar ratio (45:1) led to an improved conversion rate and the formation of the species with up to 7 dehydrations in the LC-HR-MS analysis.

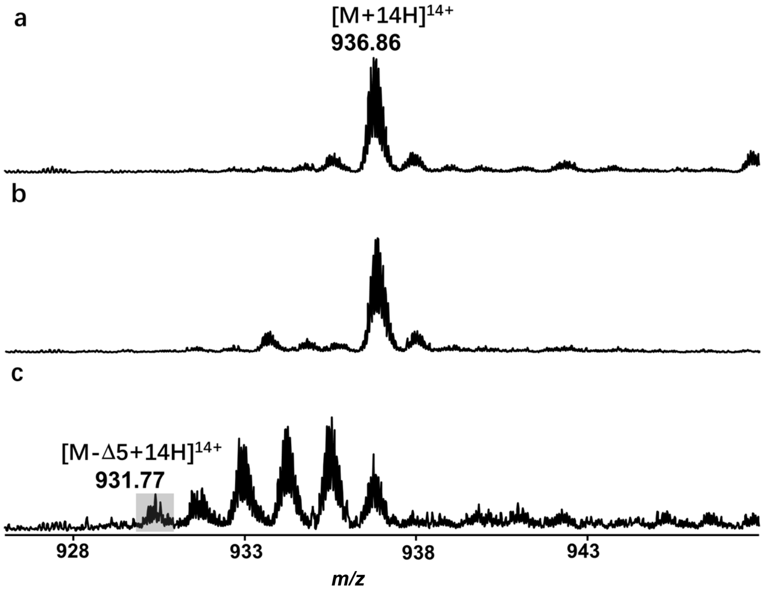

Supplementary Figure 5. HR-MS analysis showed the *in trans* activation of AMdnC to process the AMdnA with an *N*-His$_6$ tag. a AMdnA with an *N*-His$_6$ tag. **b** AMdnA with an *N*-His$_6$ tag remained unprocessed in the reaction of AMdnC. **c** Up to 5 dehydrations were observed from AMdnA with an *N*-His$_6$ tag when an equal molar of MdnA$_{9–22}$ was added to the AMdnC reaction.

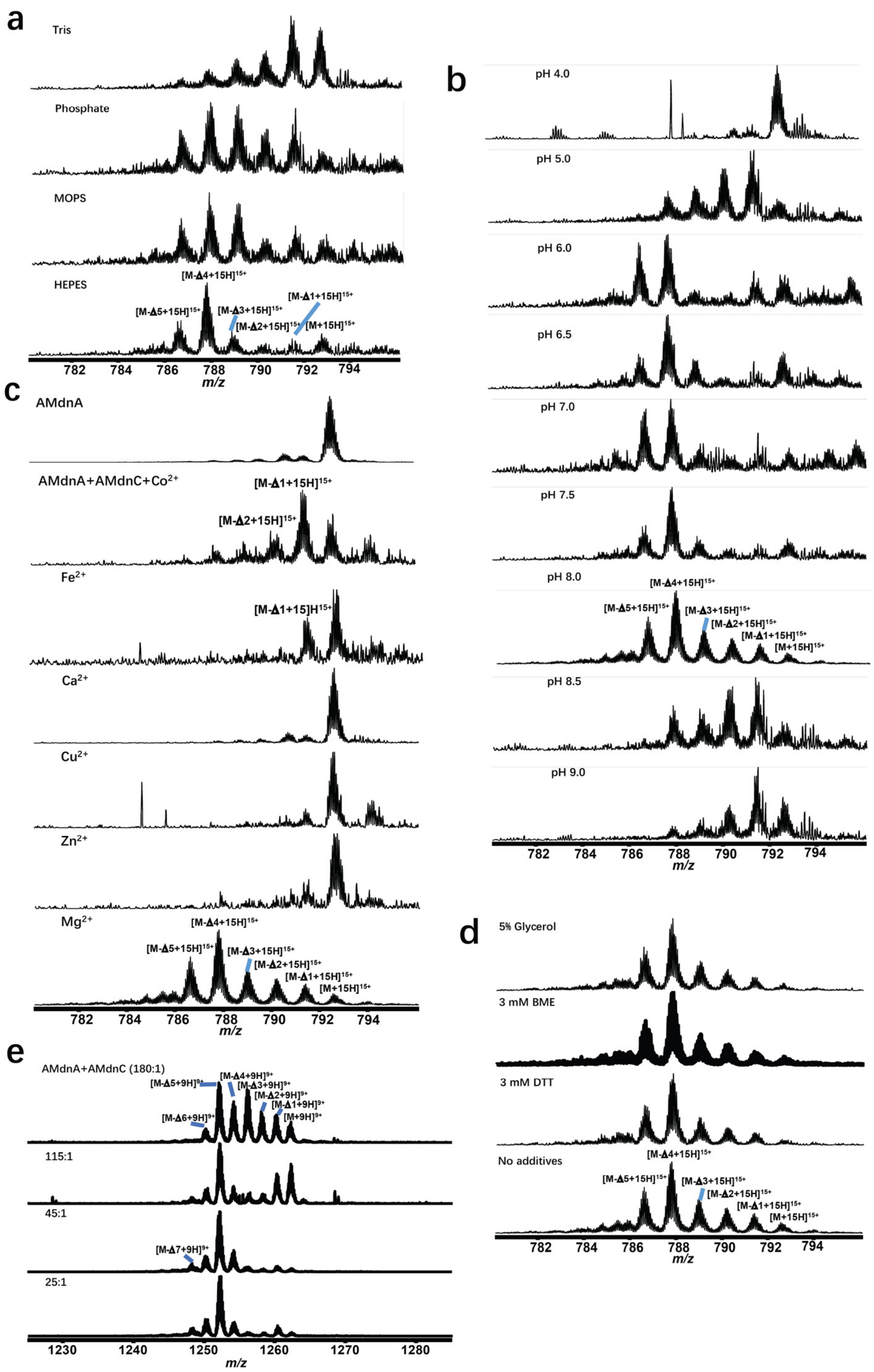

Supplementary Figure 6. **The optimization of AMdnC reaction conditions**. **a** buffers (50 mM), **b** pH, **c** divalent cations (10 mM), **d** common additives, **e** enzyme dosages were examined in the AMdnC reactions. AMdnA with a *C*-His$_6$ tag or tag-free AMdnA was used as substrate. The optimal conditions were selected based on the conversion rate, the percentage of species with a higher degree of dehydration and the stability of the substrate. Co^{2+} was accepted as co-catalytic divalent cation despite a lower conversion rate. HEPES buffer system (50 mM) with 5% glycerol, pH 8.0, 10 mM Mg^{2+} was selected as the AMdnC reaction conditions in the following experiments.

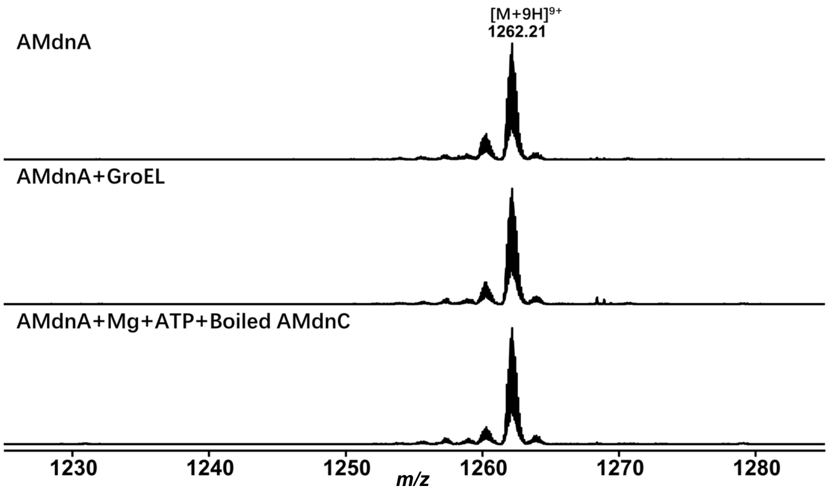

Supplementary Figure 7. LC-HR-MS analysis of negative controls of AMdnC reactions. Purified recombinant GroEL and boiled AMdnC did not process the substrate AMdnA.

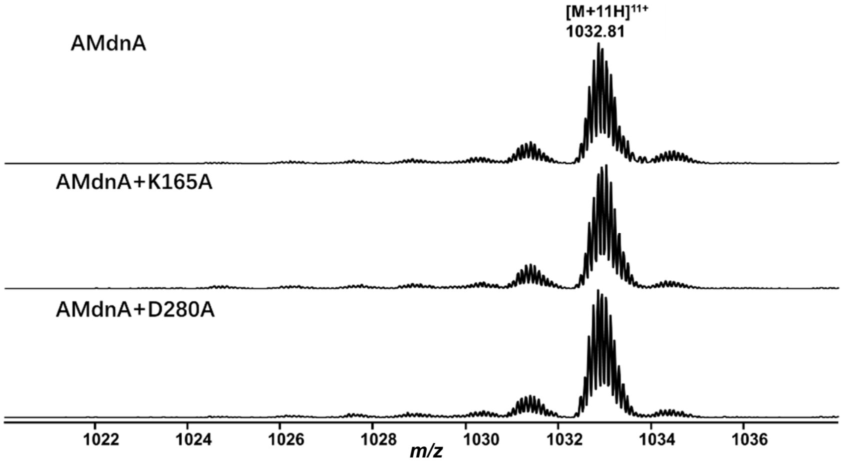

Supplementary Figure 8. AMdnC mutants K165A and D280A do not modify AMdnA. Under the standard AMdnC reaction conditions, no processed species were observed, indicating both AMdnC mutants were catalytically inactive. Importantly, copurified contaminants of AMdnC were not responsible for the observed dehydrations of AMdnA.

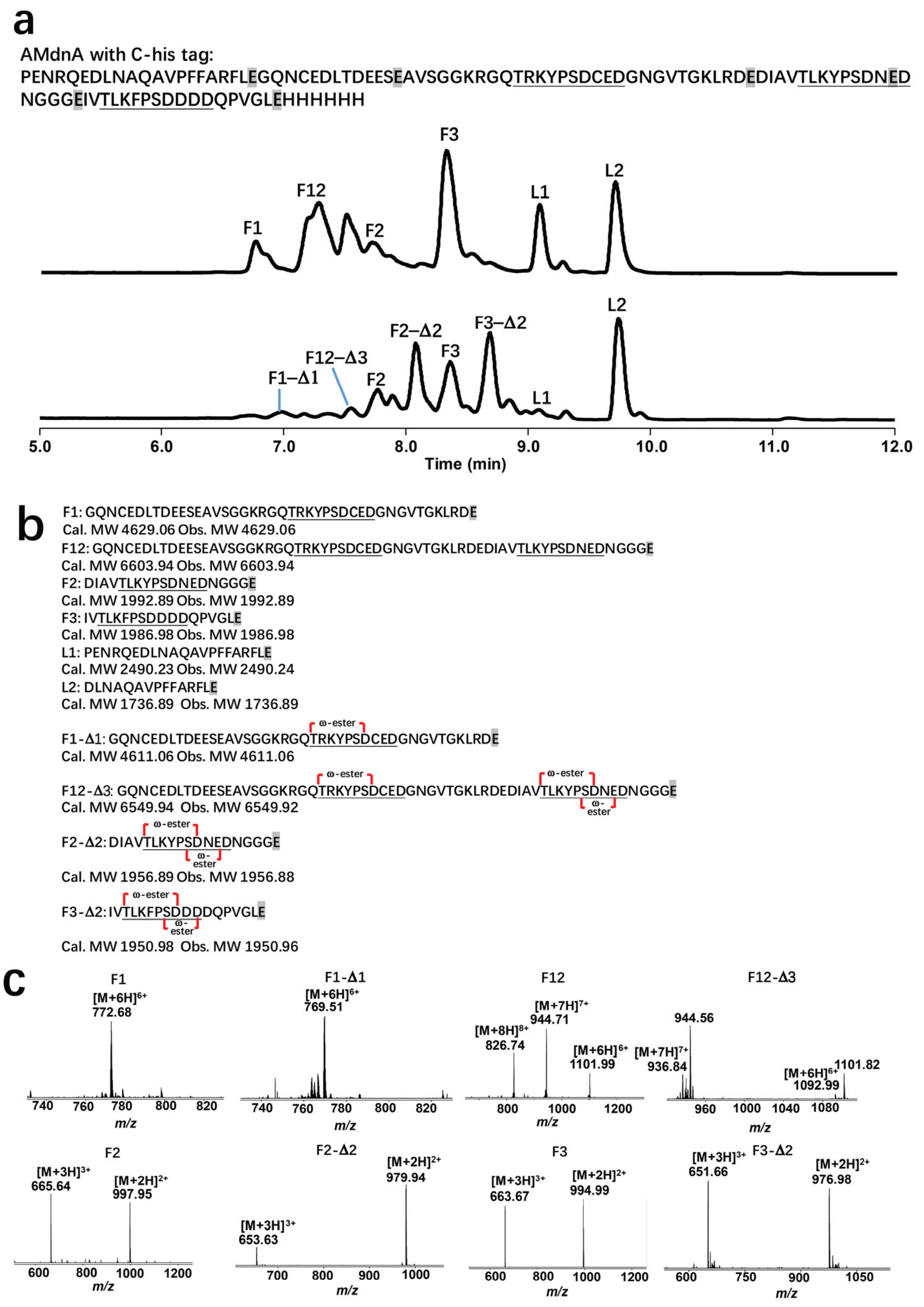

Supplementary Figure 9. AMdnC processes AMdnA with a *C*-His$_6$ tag. a HPLC traces of intact (up) and processed (down) AMdnA with a *C*-His$_6$ tag after GluC digestion. Key chromatographic peaks were labeled with the names of the corresponding peptides released by GluC. **b** The amino acid sequences, calculated and observed monoisotopic masses of each peptide released by GluC digestion. The regions of putative core peptide were underlined and the GluC digestion sites were highlighted in grey. **c** Charge numbers and the accurate mass values were labeled for key peaks in the HRMS spectra of the proteolytic peptides.

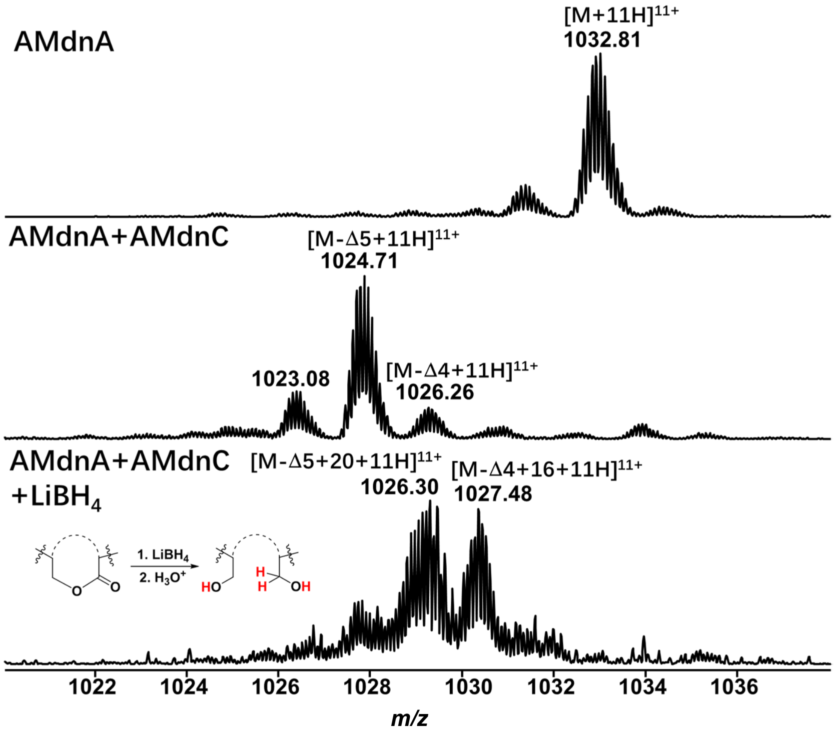

Supplementary Figure 10. Chemical reduction of the lactone bonds to quantitate the lactonization status of processed AMdnAs. Mass increase of 20 Da was observed, indicating the species with 5 dehydrations in the processed AMdnA species as substrate of the reduction reaction. The mass increase of 16 Da can be derived from the reduction of AMdnA-Δ4 or less likely, incomplete reduction of AMdnA-Δ5.

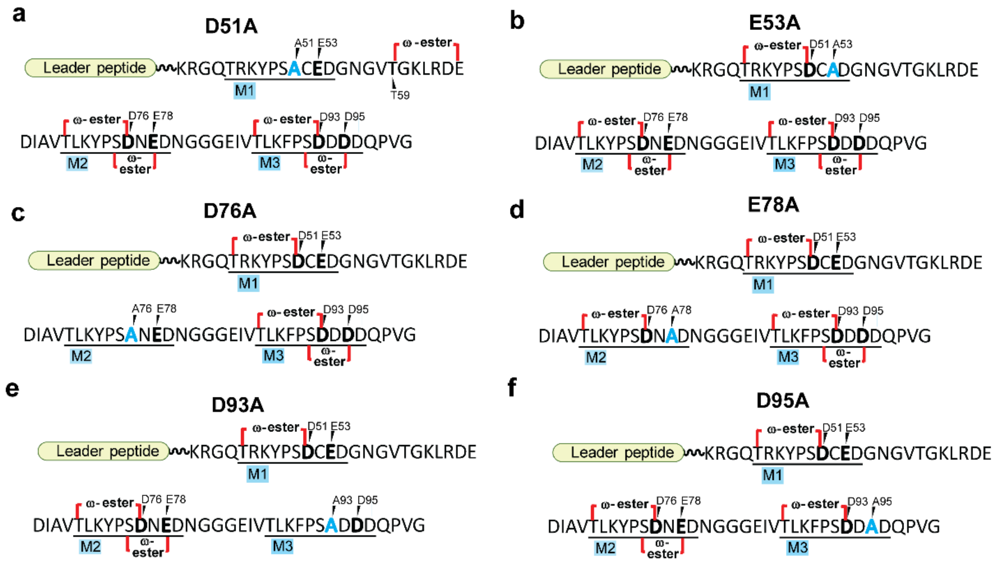

Supplementary Figure 11. Graphic representation of the structures of the most advanced AMdnA mutants in the AMdnC reactions. a AMdnA D51A, **b** E53A, **c** D76A, **d** E78A, **e** D93A, and **f** D95A were used as the substrates.

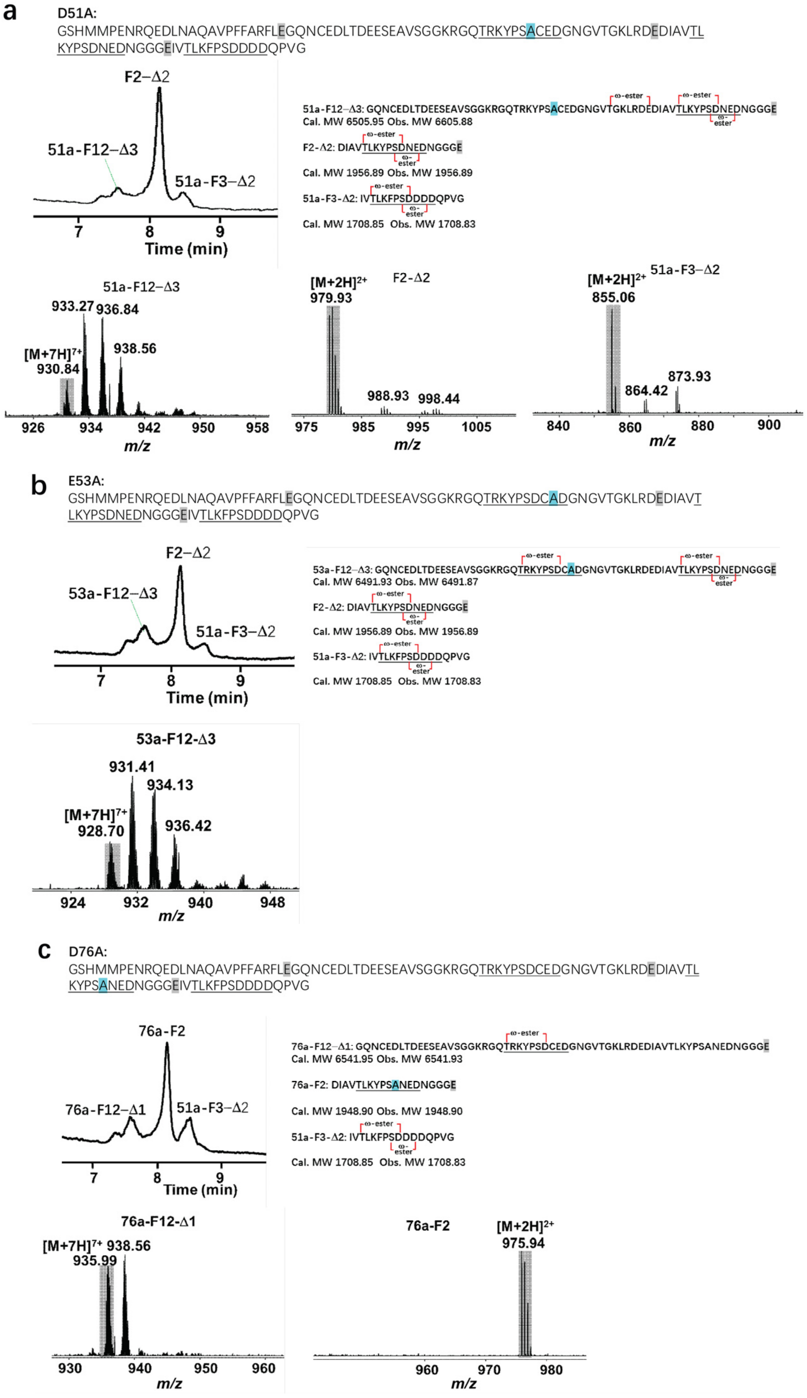
a
D51A:
GSHMMPENRQEDLNAQAVPFFARFLEGQNCEDLTDEESEAVSGGKRGQTRKYPSACEDGNGVTGKLRDEDIAVTLKYPSDNEDNGGGEIVTLKFPSDDDDQPVG
F2–Δ2
51a-F12–Δ3
51a-F3–Δ2
Time (min)
51a-F12–Δ3: GQNCEDLTDEESEAVSGGKRGQTRKYPSACEDGNGVTGKLRDEDIAVTLKYPSDNEDNGGGE
Cal. MW 6505.95 Obs. MW 6605.88
F2-Δ2: DIAVTLKYPSDNEDNGGGE
Cal. MW 1956.89 Obs. MW 1956.89
51a-F3-Δ2: IVTLKFPSDDDDQPVG
Cal. MW 1708.85 Obs. MW 1708.83
ω-ester
ester
[M+7H]7+
930.84
933.27
936.84
938.56
[M+2H]2+
979.93
988.93
998.44
855.06
864.42
873.93
m/z
b
E53A:
GSHMMPENRQEDLNAQAVPFFARFLEGQNCEDLTDEESEAVSGGKRGQTRKYPSDCADGNGVTGKLRDEDIAVTLKYPSDNEDNGGGEIVTLKFPSDDDDQPVG
53a-F12–Δ3
53a-F12–Δ3: GQNCEDLTDEESEAVSGGKRGQTRKYPSDCADGNGVTGKLRDEDIAVTLKYPSDNEDNGGGE
Cal. MW 6491.93 Obs. MW 6491.87
F2-Δ2: DIAVTLKYPSDNEDNGGGE
Cal. MW 1956.89 Obs. MW 1956.89
51a-F3-Δ2: IVTLKFPSDDDDQPVG
Cal. MW 1708.85 Obs. MW 1708.83
928.70
931.41
934.13
936.42
c
D76A:
GSHMMPENRQEDLNAQAVPFFARFLEGQNCEDLTDEESEAVSGGKRGQTRKYPSDCEDGNGVTGKLRDEDIAVTLKYPSANEDNGGGEIVTLKFPSDDDDQPVG
76a-F2
76a-F12–Δ1
76a-F12–Δ1: GQNCEDLTDEESEAVSGGKRGQTRKYPSDCEDGNGVTGKLRDEDIAVTLKYPSANEDNGGGE
Cal. MW 6541.95 Obs. MW 6541.93
76a-F2: DIAVTLKYPSANEDNGGGE
Cal. MW 1948.90 Obs. MW 1948.90
51a-F3-Δ2: IVTLKFPSDDDDQPVG
Cal. MW 1708.85 Obs. MW 1708.83
935.99
938.56
975.94

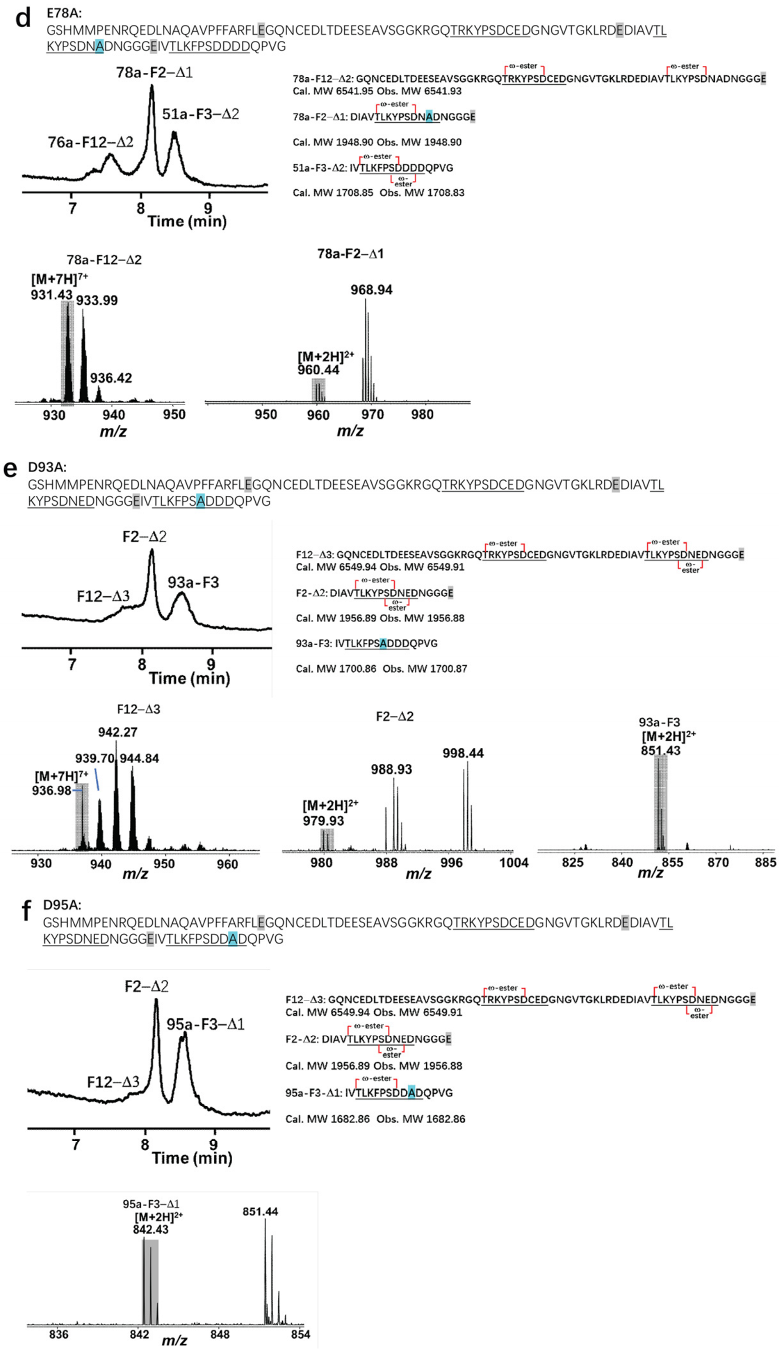

Supplementary Figure 12. Proteomic analysis of the processed AMdnA mutants. a D51A, b E53A, c D76A, d E78A, e D93A, and f D95A were used as the substrates of AMdnC reactions. The sequences, EIC of the modified core peptides, calculated and observed monoisotopic mass of each peptide released by GluC digestion were listed. The regions of putative core peptides were underlined. The GluC digestion sites and alanine substituted residues were highlighted in grey and cyan, respectively. Charge numbers and the accurate mass values were labeled for key peaks in the HRMS spectra of the proteolytic peptides. The proteolytic peptides were named using in the following formula: the mutation position-name of the WT counterpart-the number of dehydrations.

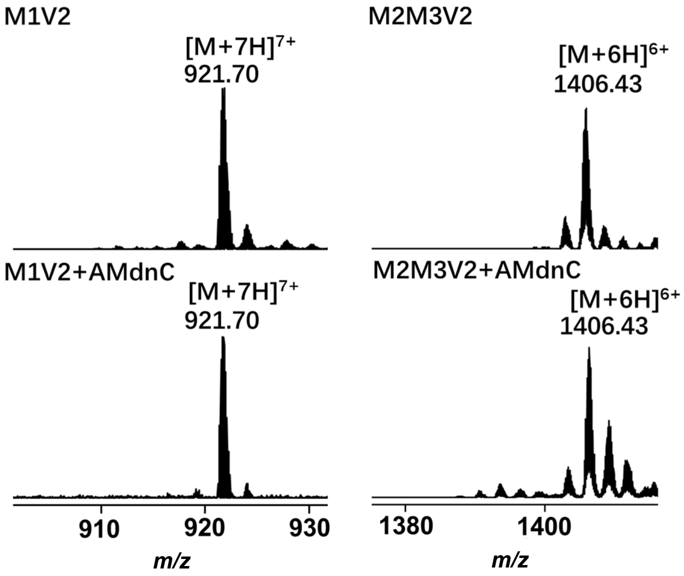

Supplementary Figure 13. AMdnC did not process AMdnA variants M1V2 and M2M3V2. These two substrates do not contain the quasi core peptide.

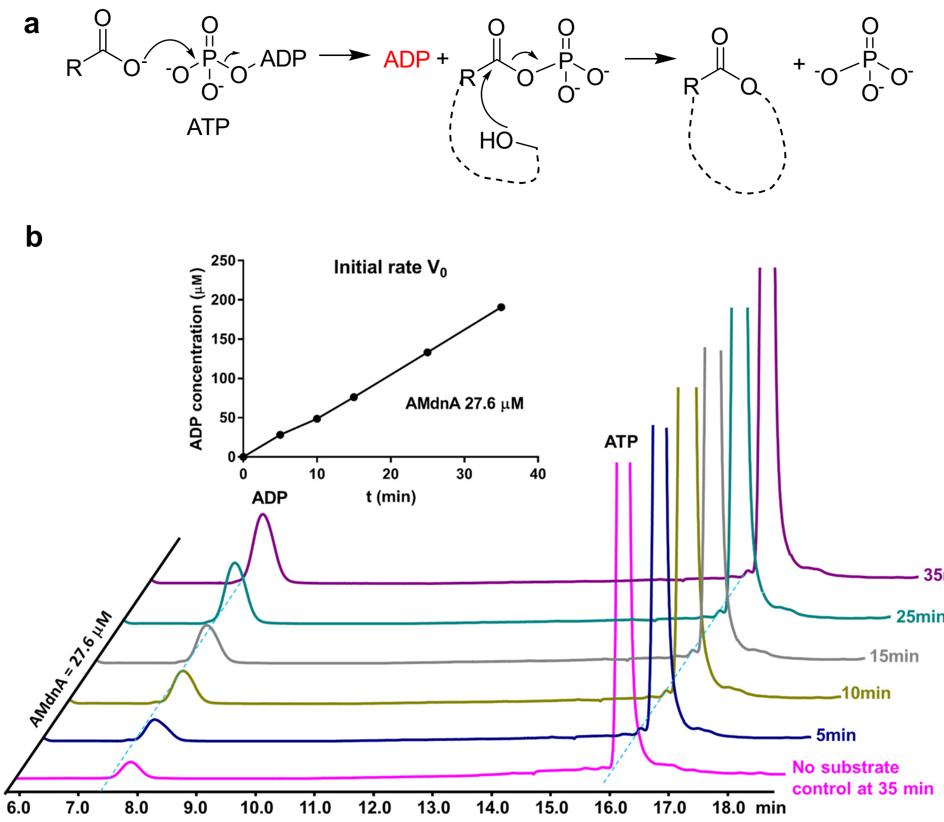

Supplementary Figure 14. Representative HPLC traces in quantitating the net production of ADP in the AMdnC reactions. a AMdnC-catalyzed macrocyclization involves the phosphorylation to release ADP and then the nucleophilic attach of activated carboxylic acid side chain to form the intramolecular lactone bond. **b** Kinetic analysis of the AMdnC reactions. Representative HPLC traces for the separation of ADP and ATP in the reaction mixtures with 27.6 μM AMdnA as the substrate, and initial rate determination were shown. The automatic ATP hydrolysis, leading to the production of ADP, in the no substrate controls at various time points was treated as background and subtracted for the calculation of initial rates.

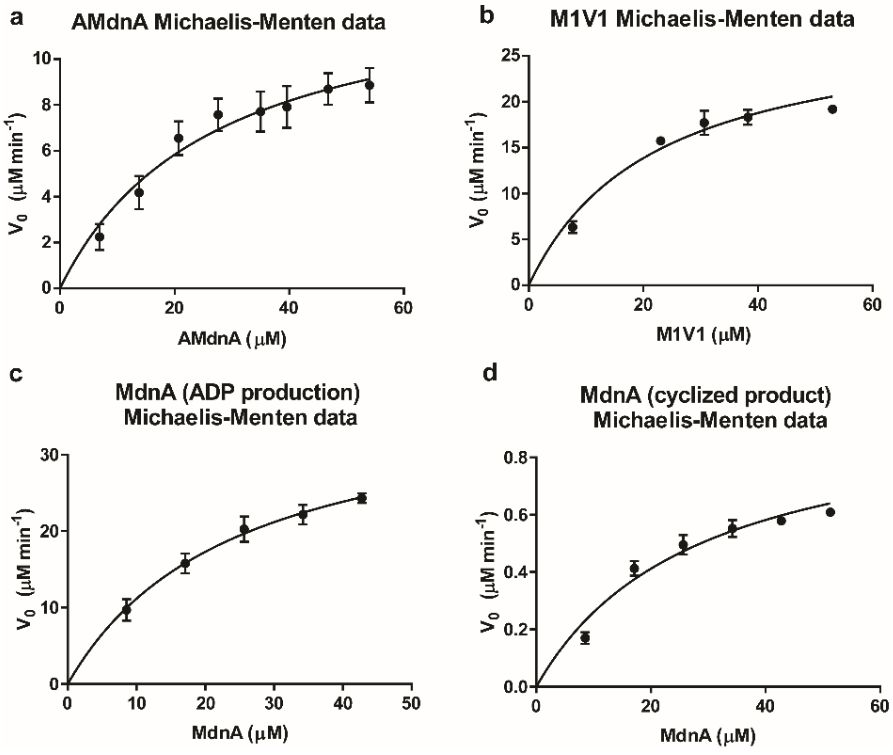

Supplementary Figure 15. Initial rates of AMdnC toward different substrates. a AMdnA, **b** M1V1, **c** MdnA, and **d** MdnA at various concentrations were used in the AMdnC reactions to create the Michaelis-Menten plots. Data represented mean ± s.d. (n =3).

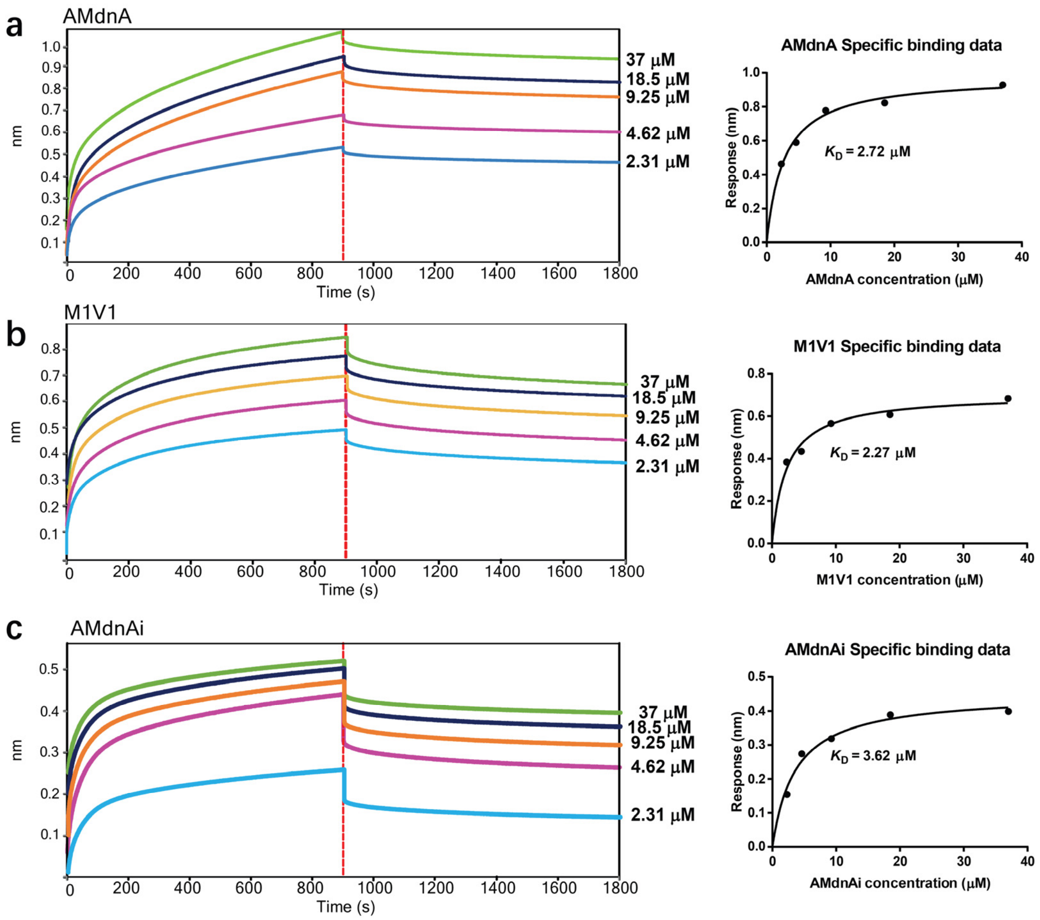

Supplementary Figure 16. Determination of binding affinities of AMdnC with AMdnA and variants. a AMdnA, **b** M1V1, and **c** AMdnAi at various concentrations were used as analytes. Sensorgrams were obtained from an Octet RED384 instrument using a series of concentrations of analytes shown in the figures. K_D values were calculated from 1:1 global fitting for AMdnA, M1V1 and AMdnAi.

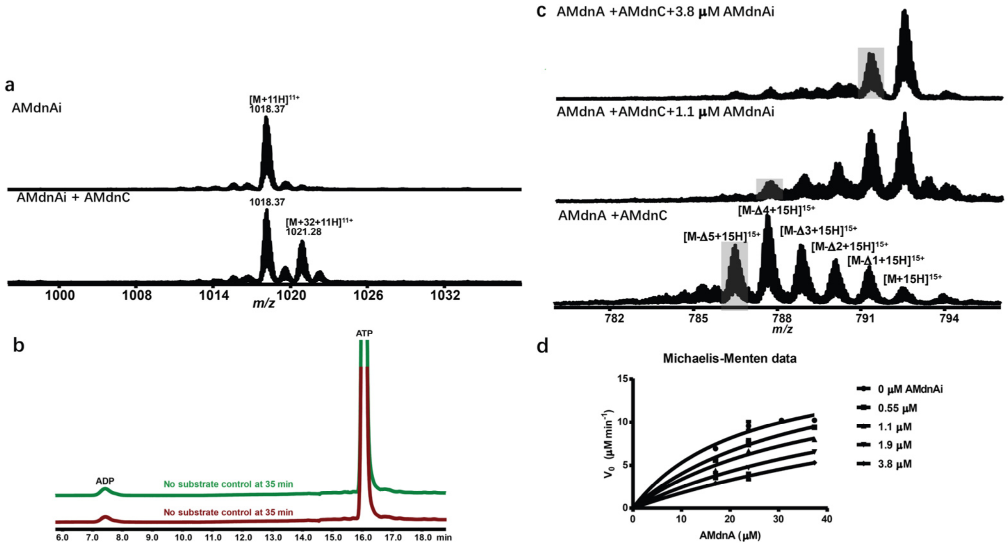

Supplementary Figure 17. AMdnAi is a competitive inhibitor of AMdnA in the AMdnC reaction. a AMdnAi stayed unchanged under the standard AMdnC reaction conditions, suggesting the abolished reactivity due to the quadruple alanine mutations. The peak with an m/z of 1021.88 represented the AMdnAi species whose cysteines both were automatically oxidized. **b** Net ADP productions in the negative control (no substrate) and in the AMdnC reaction with AMdnAi as the substrate were at the same level after the incubation under standard condition for 35 min, further indicating AMdnAi as inactive substrate of AMdnC. **c** AMdnAi inhibited the enzymatic processing of AMdnA in a dose-dependent manner. AMdnAi at 3.8 μM led to the formation of AMdnA-Δ1 as the most advanced species of the reaction, confirming that the distributive catalysis of AMdnC involves the iterative releasing and recapturing of reaction intermediates. **d** Michaelis-Menten plot created with various initial rates at different substrate and inhibitor concentrations demonstrated the competitive inhibition of AMdnC by AMdnAi. The K_i was calculated to be 1.2 ± 0.1 μM.

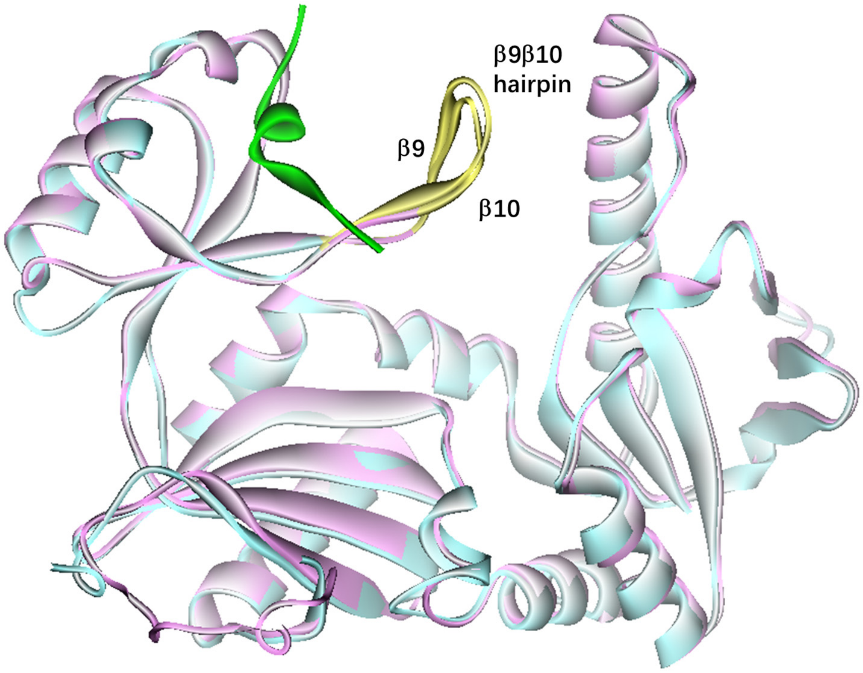

Supplementary Figure 18. Ribbon diagram of homology model of AMdnC superimposed on the crystal structure of MdnC (PDB code 5IG9).[4] AMdnC, $MdnA_{9\text{-}22}$ and MdnC were rendered in purple, green and blue, respectively. The β9β10 hairpin regions were labeled and highlighted in yellow.

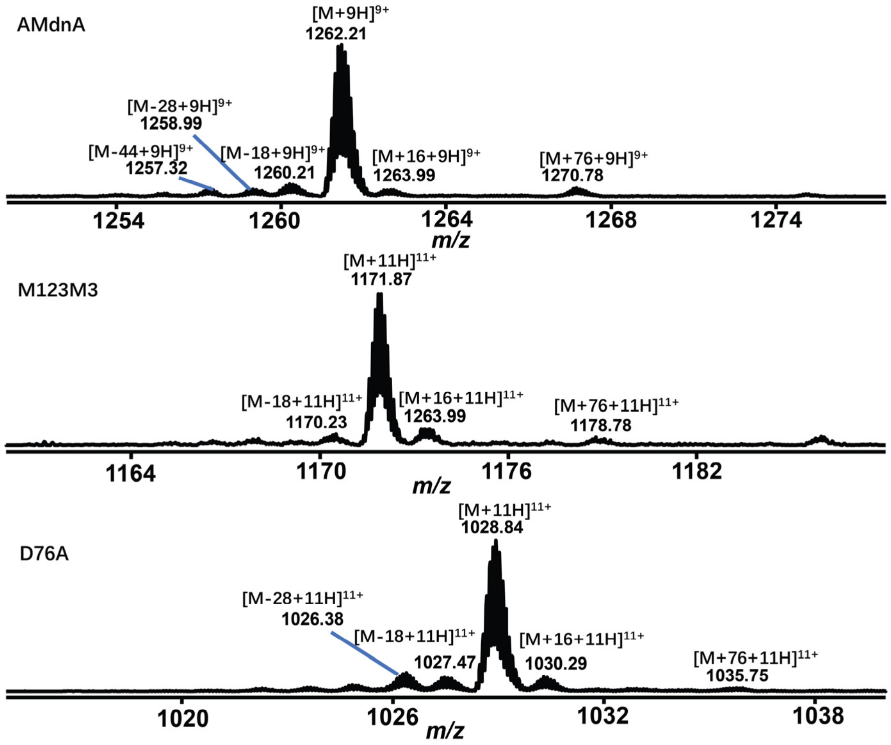

Supplementary Figure 19. *E. coli* made several modifications on recombinant AMdnA, its mutants and variants at the low levels. Putative oxidized and dehydrated species were detected, whose masses were increased by 16 Da and decreased by 18 Da compared to intact substrates, while masses of two others were decreased by 28 Da and increased by 76 Da, respectively.

Supplementary reference

1 Notredame, C., Higgins, D. G. & Heringa, J. T-Coffee: A novel method for fast and accurate multiple sequence alignment. *J. Mol. Biol.* **302**, 205-217 (2000).

2 Waterhouse, A. M., Procter, J. B., Martin, D. M. A., Clamp, M. & Barton, G. J. Jalview Version 2-a multiple sequence alignment editor and analysis workbench. *Bioinformatics* **25**, 1189-1191 (2009).

3 Bhatt, D. P., Chen, X. S., Geiger, J. D. & Rosenberger, T. A. A sensitive HPLC-based method to quantify adenine nucleotides in primary astrocyte cell cultures. *J. Chromatogr. B Analyt. Technol. Biomed. Life Sci.* **889**, 110-115 (2012).

4 Li, K., Condurso, H. L., Li, G., Ding, Y. & Bruner, S. D. Structural basis for precursor protein-directed ribosomal peptide macrocyclization. *Nat. Chem. Biol.* **12**, 973-979 (2016).
